# *Opuntia humifusa* modulates morphological changes characteristic of asthma via IL-4 and IL-13 in an asthma murine model

**DOI:** 10.1080/16546628.2017.1393307

**Published:** 2017-10-24

**Authors:** Soon-Young Lee, Chun-Sik Bae, Young-hoon Choi, Nam-Sook Seo, Chang-Su Na, Jin-Cheol Yoo, Seung Sik Cho, Dae-Hun Park

**Affiliations:** ^a^ College of Oriental Medicine, Dongshin University, Naju, Jeonnam, Korea; ^b^ College of Veterinary Medicine, Chonnam National University, Gwangju Korea; ^c^ Department of Pharmacy, College of Pharmacy, Mokpo National University, Muan, Jeonnam, Korea; ^d^ Department of Pharmacy, College of Pharmacy, Chosun University, Gwangju, Korea

**Keywords:** *Opuntia humifusa*, ovalbumin-induced asthma, IL-4, IL-13

## Abstract

Asthma is a chronic pulmonary disease that affects an estimated 235 million people worldwide, but asthma drugs have many adverse effects. *Opuntia humifusa* (eastern prickly pear) has been used as a food and traditional medicine worldwide; however, its anti-asthmatic effects have not been reported. We evaluated *O. humifusa* as a potential therapeutic or preventive component of anti-asthmatic drugs. We divided ovalbumin-sensitized mice into the following groups: normal control, asthma-induced control, dexamethasone-treated group (positive control), 50 mg/kg *O. humifusa*-treated group, 100 mg/kg *O. humifusa*-treated group, and 500 mg/kg *O. humifusa-*treated group. Levels of Th1/Th2/Th17-related cytokines were evaluated using RT-PCR, ELISA, and immunohistochemistry. *O. humifusa* dose-dependently suppressed the morphological changes typically observed in asthma, such as goblet cell hyperplasia, inflammatory cell infiltration, mucous hypersecretion, and relative basement membrane thickening in the respiratory system. These results may be attributable to regulation of Th1-/Th2-/Th17-related factors, especially interleukin (IL)-4 and IL-13. We conclude that *O. humifusa* is a potential anti-asthmatic functional food.

**Abbreviations**: *O. humifusa: Opuntia humifusa*; Th: helper T; RT-PCR: real-time polymerase chain reaction; ELISA: enzyme-linked immunosorbent assay; IL: interleukin; WHO: World Health Organization; IFN-γ: interferon gamma; TNF-α: tumor necrosis factor-alpha; IgE: immunoglobulin E; CD: cluster of differentiation; OVA: ovalbumin; DEX: dexamethasone; BALF: bronchoalveolar fluid; H&E: hematoxylin and eosin; PAS: periodic acid-schiff; PBS: phosphate-buffered saline; BM: basement membrane; cDNA: complementary deoxyribonucleic acid; RNA: ribo nucleic acid; RIPA: radioimmunoprecipitation assay; IHC: immunohistochemistry; HPLC: high-performance liquid chromatography; SD: standard deviation; WBC: white blood cells; APCs: antigen-presenting cells

## Introduction

Asthma is a chronic disease related to the pulmonary system; the number of asthma patients worldwide was estimated at 235 million in 2013. Asthma’s many inducers are referred to as allergens, and include pollen, pet dander, dust mites, tobacco smoke, and environmental pollutants [1]. Asthma is an incurable chronic disease; its typical symptoms vary from cough to obstructive apnea caused by mucous hypersecretion, epithelial hyperplasia, basement membrane thickening, and inflammatory cell infiltration near bronchioles and vessels [2,3].

It has been shown that asthma is the result of a Th1/Th2 imbalance [2]. Th1-related cytokines include interferon gamma (IFN-γ), which affects the severity of asthma [4], and interleukin 12 (IL-12), which induces Th1 cells and suppresses Th2 cells [5,6]. Th2-related cytokines include IL-4, IL-5, IL-13, and Th17-related cytokines (tumor necrosis factor-alpha (TNF-α) IL-6, and IL-1β) [7]. IL-4 modulates IgE levels, resulting in inflammatory cell migration to inter-respiratory cells [8], and IL-13 induces morphological changes in the pulmonary system typical of asthma, such as mucous hypersecretion, epithelial hyperplasia, base membrane thickening, inflammatory cell infiltration, and B cell activation [9–12]. TNF-α is produced by macrophages and is involved in the interaction between mast cells [13] and airway smooth muscle cells, a key mechanism for inducing airway hyperresponsiveness [14]. In asthma, IL-6 is upregulated in pulmonary epithelial cells by various stimuli [15] and is involved in facilitating IL-4 differentiation, downregulating Th2 cell differentiation, and promoting Th17 cell differentiation [16]. Mast cells respond to inflammatory inducers in the early phase and release TNF-α to bring on typical asthma characteristics, such as contracting smooth muscles [14], attracting neutrophils and eosinophils [17], and activating T cells [18]. IL-6 is an important factor that regulates asthma induction via CD4+ cell modulation [19].

Although inhaled corticosteroids have been commonly used for suppressing typical asthma symptoms [20], they can cause various adverse health effects such as growth defects in children [21], cataracts and glaucoma, hypertension, hyperlipidemia, peptic ulcers, myopathy, and immunological suppression [22]. These issues underscore the need to develop new anti-asthmatic drugs with few or no adverse effects.

*Opuntia humifusa* (*O. humifusa*, eastern prickly pear) has been used as a food worldwide. Recently, it has been shown to have antifungal [23] and anticancer [24,25] properties, as well as to improve insulin sensitivity [26] and increase bone density [27]. The objective of this study was to analyze the possible anti-asthmatic effects of *O. humifusa* and their mechanisms.

## Materials and methods

### *O. humifusa* preparation

*O. humifusa* leaves were provided by Jeollanamdo Wando Arboretum, in Jeonnam, Korea. A voucher specimen (MNUCSS-OH-01) was deposited at Mokpo National University (Muan, Korea). The leaves were separated for the present study. Air-dried and powdered *O. humifusa* leaves (10 g) were extracted twice with 80% ethanol (100 mL) at room temperature for 3 days. The resultant 80% ethanol solution was evaporated, dried, and stored at −50°C.

### Nutritional content

A 100 g sample of *O. humifusa* extract was analyzed for nutritional components by the Jeonnam Biofood Technology Center (Jeonnam, Korea). The extract was analyzed for carbohydrate, fat, cholesterol, and protein. All tests performed were in compliance with the standards recommended by the Association of Analytical Communities/Association of Official Agricultural Chemist and American Association of Cereal Chemists.

### Animal experiments

Using the same methods, two animal studies were conducted at different times. Eighty-four female BALB/c mice were purchased from Samtako (Osan, Korea) and divided into six groups according to treatment: (1) vehicle control (sterilized tap water), (2) ovalbumin (OVA)-induced asthma model, (3) 1 mg/kg/day dexamethasone with OVA induction, (4) 50 mg/kg/day *O. humifusa* with OVA induction, (5) 100 mg/kg/day *O. humifusa* with OVA induction, and (6) 500 mg/kg/day *O. humifusa* with OVA induction. On days 1 and 8, all mice except those used as the vehicle control were sensitized via intraperitoneal injections of 20 µg OVA (Sigma-Aldrich, St. Louis, MO, USA) and 1 mg aluminum hydroxide hydrate (Sigma-Aldrich) in 500 µL saline. From day 21 to day 25, the mice were challenged once daily with 5% OVA for 30 min using a nebulizer (3 mL/min, NE-U17, Omron, Kyoto, Japan). During the same 5-day period, the treatment groups were also treated once daily with oral doses of sterilized tap water, dexamethasone, 50 mg/kg/day *O. humifusa*, 100 mg/kg/day *O. humifusa*, or 500 mg/kg/day *O. humifusa* 1 h prior to the OVA challenge. The mice in the vehicle control group were sensitized with OVA according to the same procedure as the other groups of mice (20 µg OVA and 1 mg aluminum hydroxide hydrate in 500 µL saline), after which they were exposed to saline and aluminum hydroxide hydrate by a nebulizer for 5 consecutive days.

### Ethics statement

All experiments were approved by the Institutional Animal Care and Use Committee at Chonnam National University (Animal Study Approval No. 201 CNU IACUC-YB-R-2015-50).

### Bronchoalveolar fluid (BALF) analysis

BALF analysis was conducted as previously described [28]. The animal study was conducted twice. The first study group consisted of seven animals, with BALF analyzed in three mice, and the remaining mice (*n* = 4) used for morphological studies such as hematoxylin and eosin (H&E) staining and periodic acid-Schiff (PAS) staining. Further analyses included immunohistochemistry, real-time polymerase chain reaction (RT-PCR), and ELISAs. To confirm the results, a second animal study was conducted. One day after the final treatment, the mice were anesthetized with intraperitoneal injections of 50 mg/kg Zoletil (Virbac, Carros, France), and the tracheas were cannulated with disposable animal feeding needles. Lavages were performed with three 0.4 mL aliquots of cold phosphate-buffered saline (PBS). BALF samples were collected and immediately centrifuged at 3000 rpm for 5 min (Sorvall Legend Micro 17R, Thermo Fisher Scientific, Inc. Waltham, MA, USA). The cell pellets were resuspended in PBS for total and differential cell counts. The number of total cells and differential cells was counted with a Hemavet Multi-Species Hematology System (Drew Scientific Inc., Waterbury, CT, USA). Some animals after collecting cells and the others which were not used for cell collection were sacrificed with additional Zoletil injection. IgE levels in the serum were measured using a specific mouse IgE ELISA kit (BD Bioscience, catalog number 555248, San Jose, CA, USA) according to the manufacturer’s protocols.

### Histopathological analysis

Histopathological analysis was conducted as previously described [28]. Lung tissues were fixed in 10% (v/v) formaldehyde solution, dehydrated in a graded ethanol series (99.9%, 90%, 80%, and 70%), and embedded in paraffin. A total of eight animals from two studies were used, with eight animals per group used for histological analysis. Lung tissues were sectioned (4 µm) longitudinally and stained with H&E and PAS. Eight mice per each group were used for quantitative analysis and after the slides were analyzed with image analyzing software (NIS-Elements BR 4.50.00) all of them were blindly read by the two pathologists. At the first time one of them read the representative slide and finally the other evaluated all of them. Quantitative analysis of the morphological changes, such as goblet cell hyperplasia and inflammatory cell infiltration, was conducted using the H&E stained tissue. Mucous hypersecretion and relative basement membrane (BM) thickening were determined with the PAS stained tissue. Goblet cell hyperplasia, inflammatory cell infiltration, and mucous hypersecretion scores were evaluated from 0 (none) to 5 (severe). The score of relative BM thickening was calculated as follows:

1. BM thickness = area of BM/length of BM
2. Relative BM thickening = BM of each treatment group/BM of the control group

### RT-PCR

To evaluate changes in cDNA levels of IFN-γ, IL-12p40, IL-4, IL-13, TNF-α, and IL-6, which are related to asthma induction, RT-PCR analysis was conducted as suggested by Bustin et al. [29]. Total RNA was extracted from the lung using the RNeasy Mini Kit (Qiagen, Hilden, Germany) according to the manufacturer’s instructions. Total RNA (100 ng) was used as a template for the reaction. Primers were synthesized for RT-PCR as follows: IFN-γ forward 5′-GGCCATCAGCAACAACATAAG-3′, IFN-γ reverse 5′-GTTGACCTCAAACTTGGCAATAC-3′; IL-12p40 forward 5′-GGACCAAAGGGACTATGAGAAG-3′, IL-12p40 reverse 5′-CTTCCAACGCCAGTTCAATG-3′; IL-4 forward 5′-ACAGGAGAAGGGACGCCAT-3′, IL-4 reverse 5′-GAAGCCCTACAGACGAGCTCA-3′; IL-13 forward 5′-CAGCCCTCAGCCATGAAATA-3′, IL-13 reverse 5′-CTTGAGTGTGTAACAGGCCATTCT-3′; IL-6 forward 5′-GATAAGCTGGAGTCACAGAAGG-3′, IL-6 reverse 5′-TTGCCGAGTAGATCTCAAAGTG-3′; TNF-α forward 5′-CTGAGTTCTGCAAAGGGAGAG-3′, TNF-α reverse 5′-CCTCAGGGAAGAATCTGGAAAG-3′; GAPDH forward 5′-GTGGAGTCATACTGAACATGTAG-3′, GAPDH reverse 5′-AATGGTGAAGGTCGGTGTG-3′. The RT-PCR cycles consisted of denaturation at 95°C for 5 s, and annealing/extension at 65°C for 30 s for 40 cycles.

### ELISA

To analyze the levels of IFN-γ, IL-12p40, IL-4, IL-6, and TNF-α in lung tissue, OptEIA mouse ELISAs were purchased from BD Biosciences. IL-13 levels were assessed using the AbFrontier Cymax mouse ELISA kit (AbFrontier, Seoul, Korea). All assays were conducted according to the manufacturers’ guidelines. All lung samples were prepared by lysis buffer made with a protease inhibitor cocktail and RIPA buffer (Thermo Fisher Scientific). Aliquots of lung tissue from all groups were weighed and homogenized with lysis buffer. They were centrifuged at 8200 rpm for 15 min, and the supernatants were harvested and measured using a microplate reader (EZ Read 400, Biochrom, Cambourne, UK).

### IHC analysis

IHC analysis was conducted as previously described [28]. Deparaffinized tissue sections were treated with 3% hydrogen peroxide in methanol for 10 min to remove endogenous peroxidase. Antigen retrieval was carried out with sodium citrate buffer (0.1 M) using the microwave method. The slides were incubated with normal serum to block nonspecific binding and then incubated for 1 h with primary antibodies (diluted 1:100 to 1:200) such as IFN-γ (sc-74104, Santa Cruz Biotechnology, Dallas, TX, USA), IL-12p40 (sc-57258), IL-4 (sc-73318), IL-13 (sc-1776), TNF-α (MyBioSource, San Diego, CA, USA), and IL-6 (Novus Biologicals, Littleton, CO, USA). The slides were incubated for 10 min with biotinylated secondary antibodies (Vector Laboratories, PK-7800, Burlingame, CA, USA) and horseradish peroxidase-conjugated streptavidin. Signals were detected using the 3,3-diaminobenzidine tetrahydrochloride substrate chromogen solution, and the cells were counterstained with Mayer’s hematoxylin.

### Constituent profiling by high-performance liquid chromatography (HPLC)

All analyses were performed using an Alliance 2695 HPLC system (Waters; Milford, MA, USA) equipped with a photodiode array detector. The analytical column was an Agilent Zorbax Extend-C18 (5 µm, 150 mm × 5 mm) with a mobile phase consisting of solvent A (acetonitrile) and solvent B (water containing 0.2% phosphoric acid) employing gradient elution (from 10/90 to 100/0, v/v) at a flow rate of 0.5 mL/min (Table 1). The column temperature was maintained at 25°C, and the detection wavelength was set at 270 nm for rutin and quercetin. The solvent was filtered through a 0.22-µm filter and degassed. The sample injection volume was 10 µL.

**Table 1.** Analytical conditions of HPLC for analysis of *O. humifusa.*

Parameters	Conditions		
Column	Zorbax extended-C18 (C18, 4.6 mm × 150 mm, 5 µm)		
Flow rate	1 mL/min		
Injection Volume	10 μL		
UV detection	270 nm		
Run time	30 min		
	Time (min)	Acetonitrile (%)	0.2% phosphoric acid (%)
	0	10	90
Gradient	7	10	90
	18	100	0
	19	10	90
	30	10	90

### Statistical analysis

Results are expressed as means ± standard deviation (SD). Group differences were evaluated by one-way analysis of variance, followed by Dunnett’s multiple comparisons test. A *p* value of <0.01 or <0.05 was considered statistically significant.

## Results

### *O. humifusa* extract has 3.9% pharmaceutical active compounds

The nutritional composition of the extract is shown in Table 2. The extract contains 24% of the recommended daily allowance (RDA) of carbohydrate (77.8 g/100 g), 9% of the RDA of protein (11.5 g/100 g), 2% of the RDA of fat (4.9 g/100 g), and 1% of the RDA of cholesterol (1.9 g/100 g).

**Table 2.** Analysis of nutritional components in the *O. humifusa* extract.

Components	Values (units/100 g)	RDA (%)^a^
Energy (kcal)	342.5	
Carbohydrate (g)	77.8	330
Protein (g)	11.5	55
Fat (g)	4.9	50
Cholesterol (mg)	1.9	300

^a^RDA, recommended daily allowance.

The total percentage of carbohydrate, protein, and fat in the extracts was 96.1%, and the chemical substances that contain pharmaceutically active compounds were estimated to be 3.9%. The extracts contained the active compound such as rutin (0.38%) and quercetin (0.1%). The two active compounds accounted for about 12.3% of the total chemical composition and are thought to have anti-asthmatic efficacy along with undetermined components.

### *O. humifusa* inhibited white blood cell proliferation and suppressed neutrophil levels

**Figure 1.**
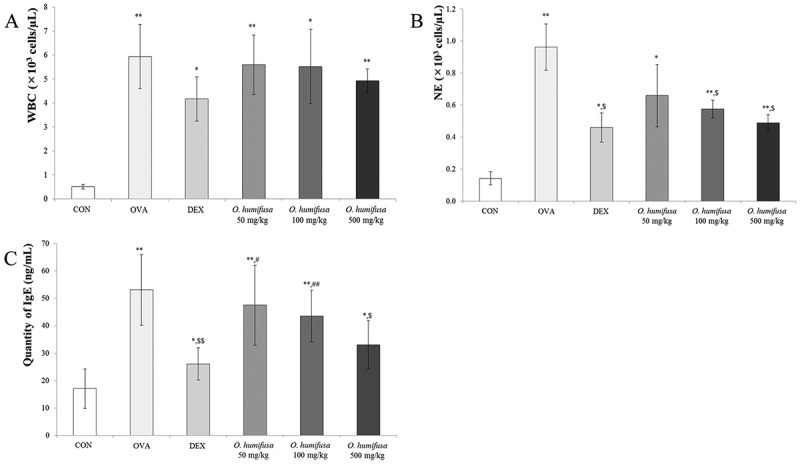
*O. humifusa* decreased the number of white blood cells (WBCs) and dose-dependently suppressed neutrophils and IgE in bronchoalveolar lavage fluid (BALF) from ovalbumin (OVA)-induced asthma in mice. (a) Although the differences were not significant, *O. humifusa* decreased WBC counts in BALF. (b) *O. humifusa* dose-dependently suppressed neutrophils in BALF following OVA treatment. (c) Similar to neutrophils, *O. humifusa* dose-dependently attenuated OVA-induced increases in IgE levels. Each bar represents the means ± SD (*N* = 6). *p < 0.05 vs. control; **p < 0.001 vs. control; ^$^ *p* < 0.05 vs. asthma induction; ^$$^ *p* < 0.01 vs. asthma induction; ^#^ *p* < 0.05 vs. dexamethasone; ^##^ *p* < 0.01 vs. dexamethasone.

The number of white blood cells (WBC) was significantly higher (Figure 1(a )) in the OVA-induced asthmatic group than that in the control group in which the vehicle was orally administered and not inhaled. Dexamethasone, which is commonly used as an asthma drug, suppressed OVA-induced increases in WBCs. Similarly, *O. humifusa* dose-dependently modulated OVA-induced increases in WBCs. Increased eosinophils in the bronchoalveolar fluid (BALF) is commonly observed in asthma patients; however, an increase in neutrophils is occasionally observed [30]. OVA increased the number of neutrophils, whereas they were suppressed by dexamethasone (Figure 1(b )); *O. humifusa* suppressed the level of neutrophils in a dose-dependent manner. Neutrophil counts of the 500 mg/kg *O. humifusa*-treated group were similar to those of the dexamethasone-treated group. Likewise, changes in IgE levels were similar to the observed changes in WBC count (Figure 1(a,c)). OVA-induced increases in IgE were suppressed by dexamethasone and *O. humifusa* treatment. Specifically, 500 mg/kg *O. humifusa* treatment significantly suppressed IgE levels; there were nonsignificant decreases in IgE levels in OVA-sensitized mice treated with 250 mg/kg *O. humifusa* and 50 mg/kg *O. humifusa*.

### *O. humifusa* significantly suppressed morphological changes in OVA-induced asthma

**Figure 2.**
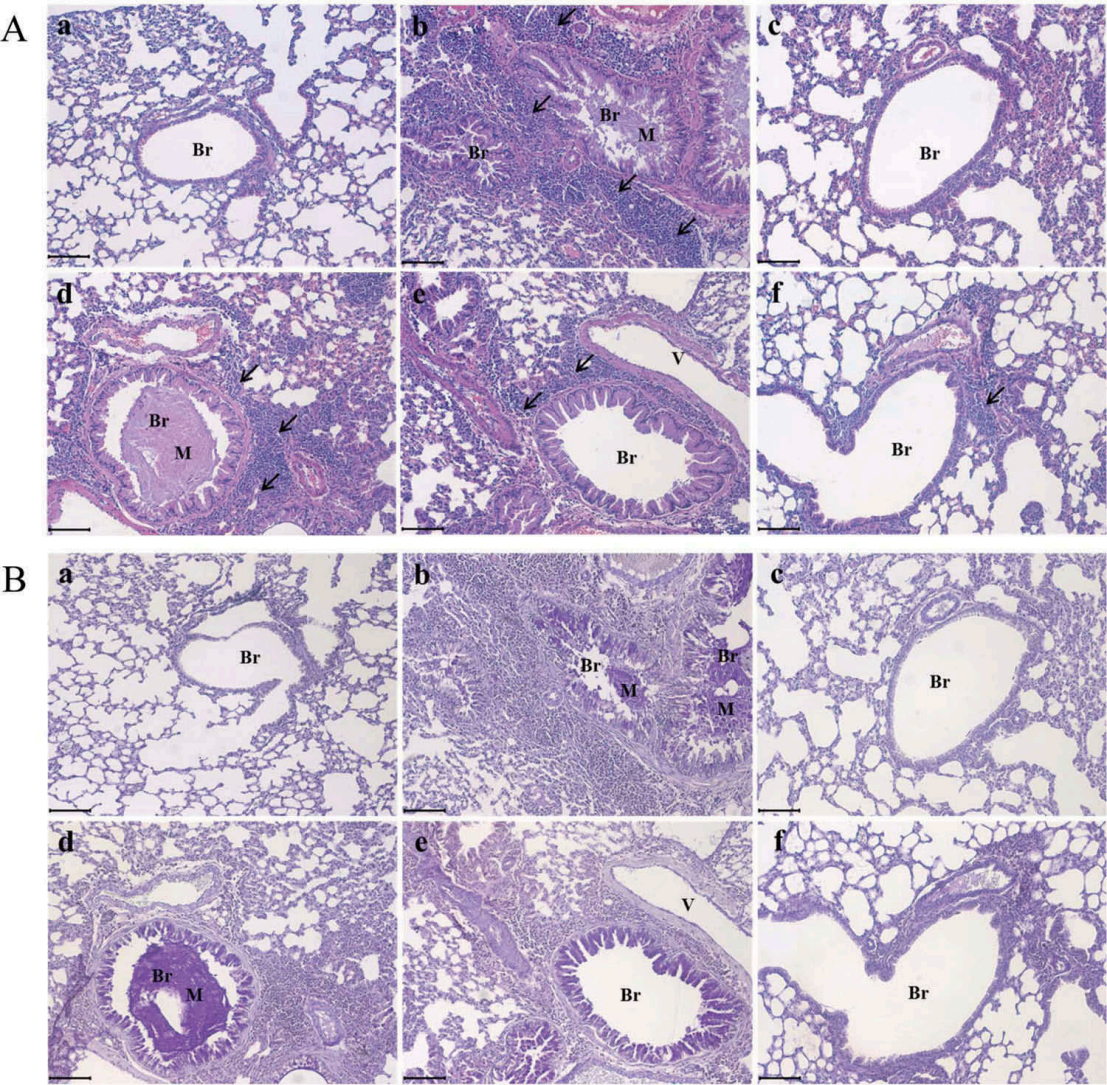
*O. humifusa* dose-dependently suppressed typical morphological changes induced by OVA treatment in the respiratory system. (a) In the H&E-stained lung, *O. humifusa* significantly modulated asthmatic changes, such as mucous hypersecretion (M), inflammatory cell infiltration in the intercellular space (arrows), epithelial cell hyperplasia, and airway remodeling. (b) In the PAS stained lung, *O. humifusa* prevented mucous secretion in a dose-dependent manner. *N* = 8. Scale Bar: 100 µm. Br, bronchiole; V, vessel; M, mucous; arrow, inflammatory cell; a, vehicle control; b, asthma induction; c, dexamethasone; d, 50 mg/kg/day *O. humifusa*; e, 100 mg/kg/day *O. humifusa*; f, 500 mg/kg/day *O. humifusa.*

OVA treatment caused morphological changes in lung tissue that were typical of asthma, including inflammatory cell infiltration near bronchioles or vessels (arrows in Figure 2(Ab)), mucous hypersecretion (M in Figure 2(Ab,Bb)), and bronchial epithelial cell hyperplasia (Figure 2(Aa,Ba)). Dexamethasone restored OVA-induced lung damage (Figure 2(Ac,2Bc)) to that observed in the lungs with normal morphology. We further confirmed the changes in OVA-induced lung damage after treatment with dexamethasone or *O. humifusa* treatment by quantitative assessments (Table 3). Goblet cell hyperplasia was only observed in the OVA- and 50–100 mg/kg *O. humifusa*-treated groups. The inflammatory cell infiltration score for the OVA-treated group was 2, whereas the scores of other treatment groups decreased to 1. Inflammatory cell infiltration was not observed in the control or dexamethasone-treated groups. The inflammatory cell infiltration score reached as high as 5 in the OVA-sensitized mice, an effect that was dose-dependently decreased by *O. humifusa* treatment. Specifically, mice treated with 50, 100, and 500 mg/kg *O. humifusa* had a score of 4, 3, and 2, respectively. Mucous hypersecretion was defined in the same manner as that of goblet cell hyperplasia or inflammatory cell infiltration (from 0 (none) to 5 (severe)). Mucous hypersecretion was observed only in the OVA and 50 g/kg *O. humifusa* treatment groups. These results suggest that OVA-induced mucous hypersecretion is suppressed by dexamethasone or *O. humifusa* treatment at doses greater than 100 mg/kg. The results of experiments measuring relative BM thickening were very similar to those of the inflammatory cell infiltration experiments; ovalbumin treatment induced relative thickening of the BM, an effect that was dose-dependently attenuated by *O. humifusa*.

**Table 3.** Quantitative results of histopatholgoical changes.

	Goblet cell hyperplasia (0–5)	Inflammatory cell infiltration (0–5)	Mucous hypersecretion (0–5)	Relative basement membrane thickening
CON	0 ± 0.3	0 ± 0.5	0 ± 0.4	1.00 ± 0.05
OVA	2 ± 0.5*	5 ± 0.9**	5 ± 0.6**	1.11 ± 0.08*
DEX	0 ± 0.5^$^	0 ± 0.5^$$^	0 ± 0.5^$$^	0.82 ± 0.09^$^
*O. humifusa* 50 mg/kg	1 ± 0.4*,^#^	4 ± 0.8**,^#^	5 ± 0.8**,^##^	0.94 ± 0.04
*O. humifusa* 100 mg/kg	1 ± 0.5*,^$^	3 ± 0.4*, ^#^	0 ± 0.3^$$^	0.90 ± 0.07^$^
*O. humifusa* 500 mg/kg	0 ± 0.4^$^	2 ± 0.8*,^$,#^	0 ± 0.5^$$^	0.88 ± 0.04^$^

Each bar represents the means ± SD (*N* = 8).

*p < 0.05 vs. control; **p < 0.001 vs. control; ^$^ *p* < 0.05 vs. asthma induction; ^$$^ *p* < 0.01 vs. asthma induction; ^#^ *p* < 0.05 vs. dexamethasone; ^##^ *p* < 0.01 vs. dexamethasone.

### *O. humifusa* decreased Th1-related cytokines such as IFN-γ and IL-12

**Figure 3.**
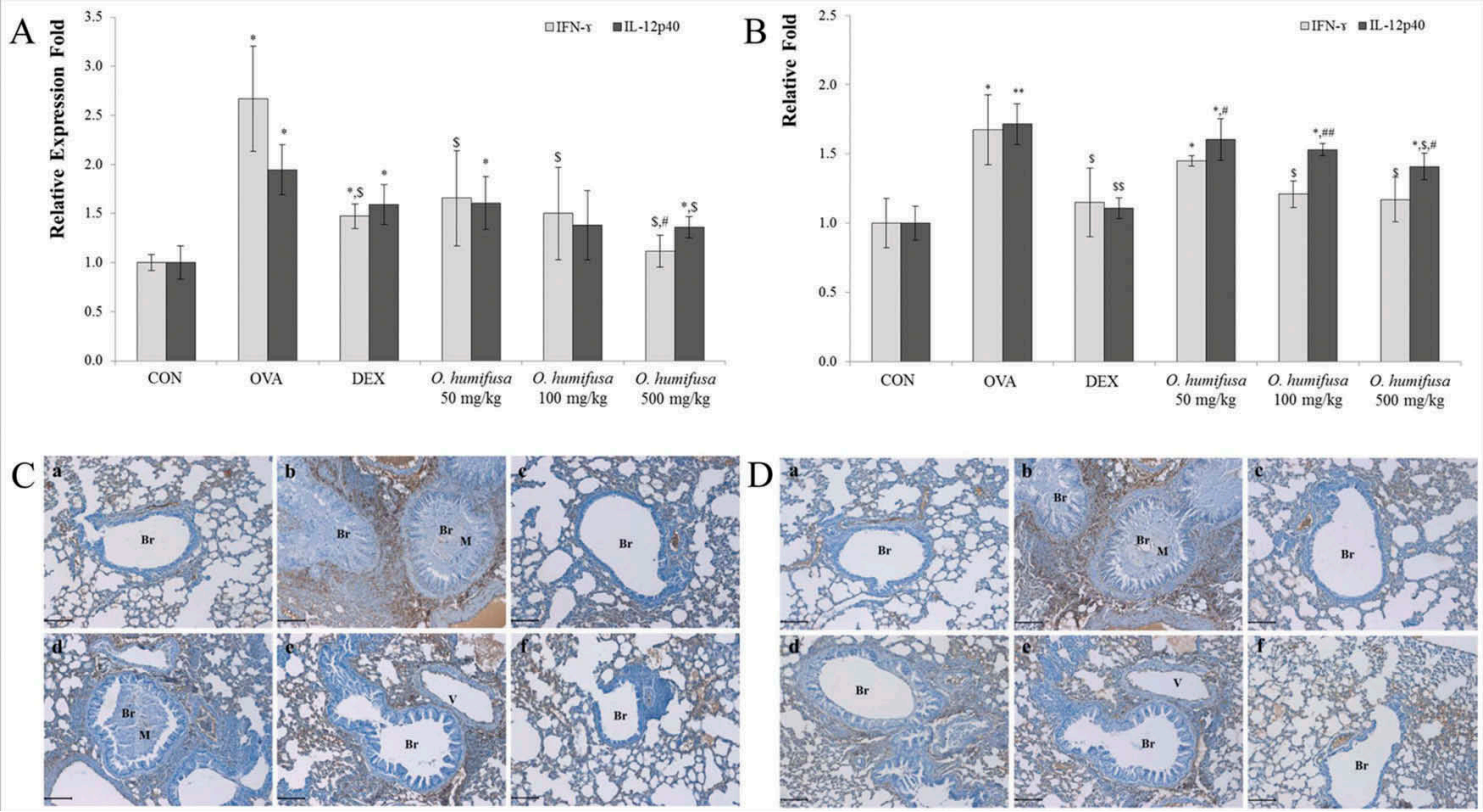
*O. humifusa* modulated gene expression and protein levels of Th1-related cytokines such as IFN-γ and IL-12p40. *O. humifusa* suppressed not only *IFN-γ* gene expression as quantified by RT-PCR (A), but also IFN-γ protein levels, as assessed by ELISA (B) and IHC (C). *O. humifusa* modulated *IL-12* gene expression, as confirmed by RT-PCR (A) and significantly inhibited the protein expression of IL-12p40, as verified by ELISA (B) and IHC (D). *N* = 8. Scale Bar: 100 µm. Br, bronchiole; V, vessel; M, mucous; a, vehicle control; b, asthma induction; c, dexamethasone; d, 50 mg/kg/day *O. humifusa*; e, 100 mg/kg/day *O. humifusa*; f, 500 mg/kg/day *O. humifusa*. *p < 0.05 vs. control; **p < 0.001 vs. control; ^$^ *p* < 0.05 vs. asthma induction; ^$$^ *p* < 0.01 vs. asthma induction; ^#^ *p* < 0.05 vs. dexamethasone; ^##^ *p* < 0.01 vs. dexamethasone.

To evaluate changes in Th1-related cytokines such as IFN-γ and IL-12, we measured gene expression levels by RT-PCR and proteins levels by enzyme-linked immunoassay (ELISA) and immunohistochemistry (IHC). OVA upregulated IL-12 gene expression (Figure 3(a)). Although there were no statistical differences, *O. humifusa* slightly suppressed the levels of IL-12 (Figure 3(b,c)). IFN-γ gene expression was upregulated by OVA treatment; however, dexamethasone and *O. humifusa* suppressed IFN-γ gene expression at all concentrations tested (Figure 3(a )). IFN-γ protein levels were dose-dependently modulated by *O. humifusa* (Figure 3(b,d)).

### *O. humifusa* dose-dependently suppressed the levels of the Th2-related cytokines IL-4 and IL-13, but not IL-5

**Figure 4.**
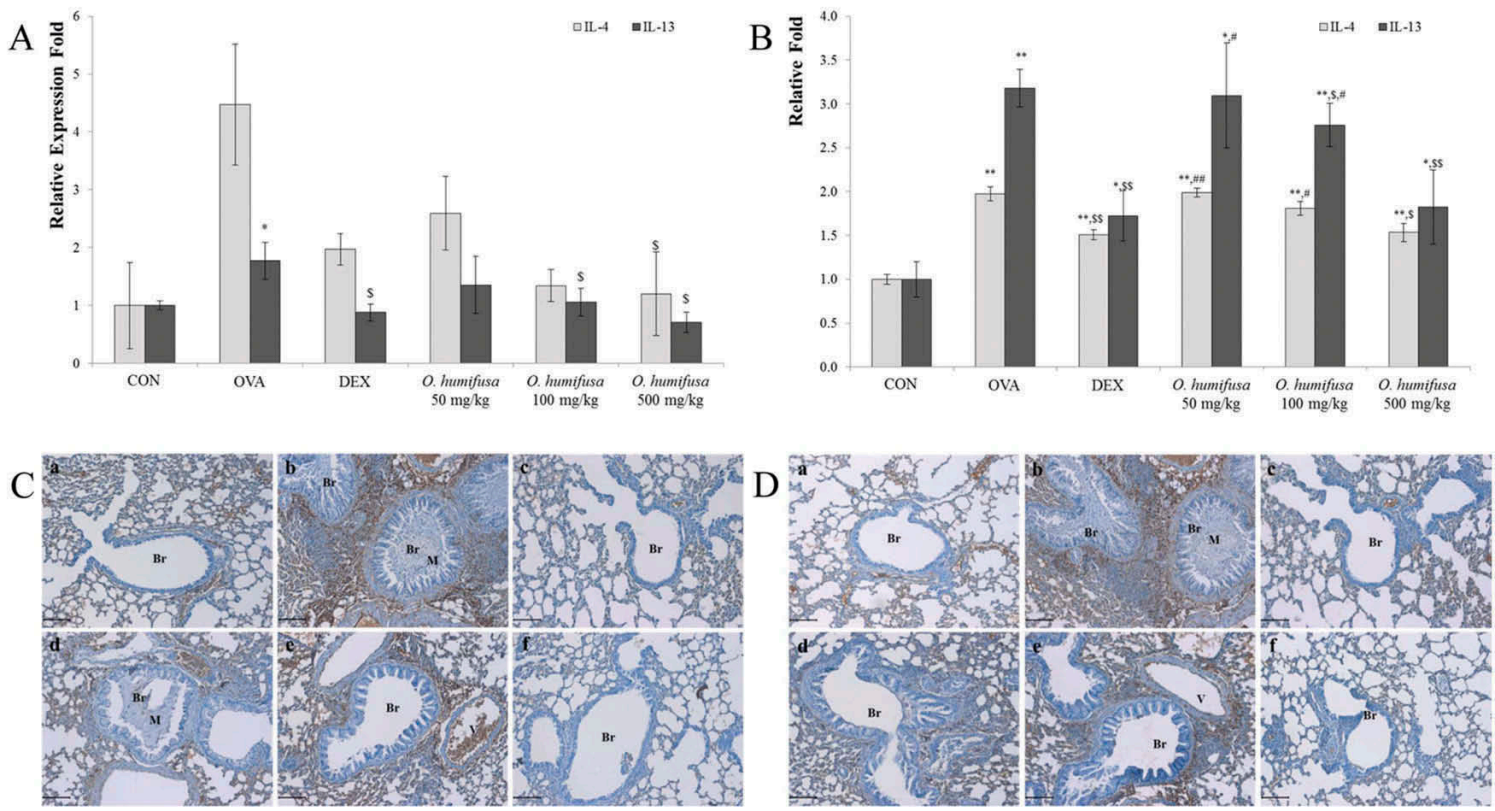
*O. humifusa* significantly suppressed Th2-related cytokines such as IL-4 and IL-13. Although *O. humifusa* downregulated gene expression levels of *IL-4*, we found no statistical differences when using RT-PCR (A); *O. humifusa* significantly suppressed *IL-4* gene expression as assessed by ELISA (B) and IHC (C). Similarly, *IL-13* gene expression levels (A) and protein levels (B, D) were dose-dependently decreased by *O. humifusa* treatments. N = 8. Scale bar: 100 µm. Br, bronchiole; V, vessel; M, mucous; a, vehicle control; b, asthma induction; c, dexamethasone; d, 50 mg/kg/day *O. humifusa*; e, 100 mg/kg/day *O. humifusa*; f, 500 mg/kg/day *O. humifusa*. *p < 0.05 vs. control; **p < 0.001 vs. control; ^$^ *p* < 0.05 vs. asthma induction; ^$$^ *p* < 0.01 vs. asthma induction; ^#^ *p* < 0.05 vs. dexamethasone; ^##^ *p* < 0.01 vs. dexamethasone.

Expression of both IL-4 and IL-13 genes was dose-dependently downregulated by *O. humifusa* treatment, whereas their expression was upregulated after treatment with ovalbumin (Figure 4(a )). Expression profiles in the 500 mg/kg *O. humifusa* treatment group were lower than those in the dexamethasone treatment group; changes in IL-4 and IL-13 protein levels followed a similar pattern (Figure 4(b–d)). Protein levels increased following dexamethasone treatment and decreased in a dose-dependent manner following *O. humifusa* treatment. IL-4 and IL-14 gene expression and protein levels were significantly regulated by *O. humifusa* treatments. Therefore, IL-4 and IL-13 may be key factors in suppressing the induction of asthma. IL-5, however, they were not modulated by *O. humifusa* treatment (data not shown). IL-4 and IL-13 promote IgE production [31]; changes in IgE levels (Figure 1(c )) reflected those of IL-4 and IL-13 (Figure 4).

### *O. humifusa* dose-dependently decreased the Th17-related cytokines TNF-α and IL-6

**Figure 5.**
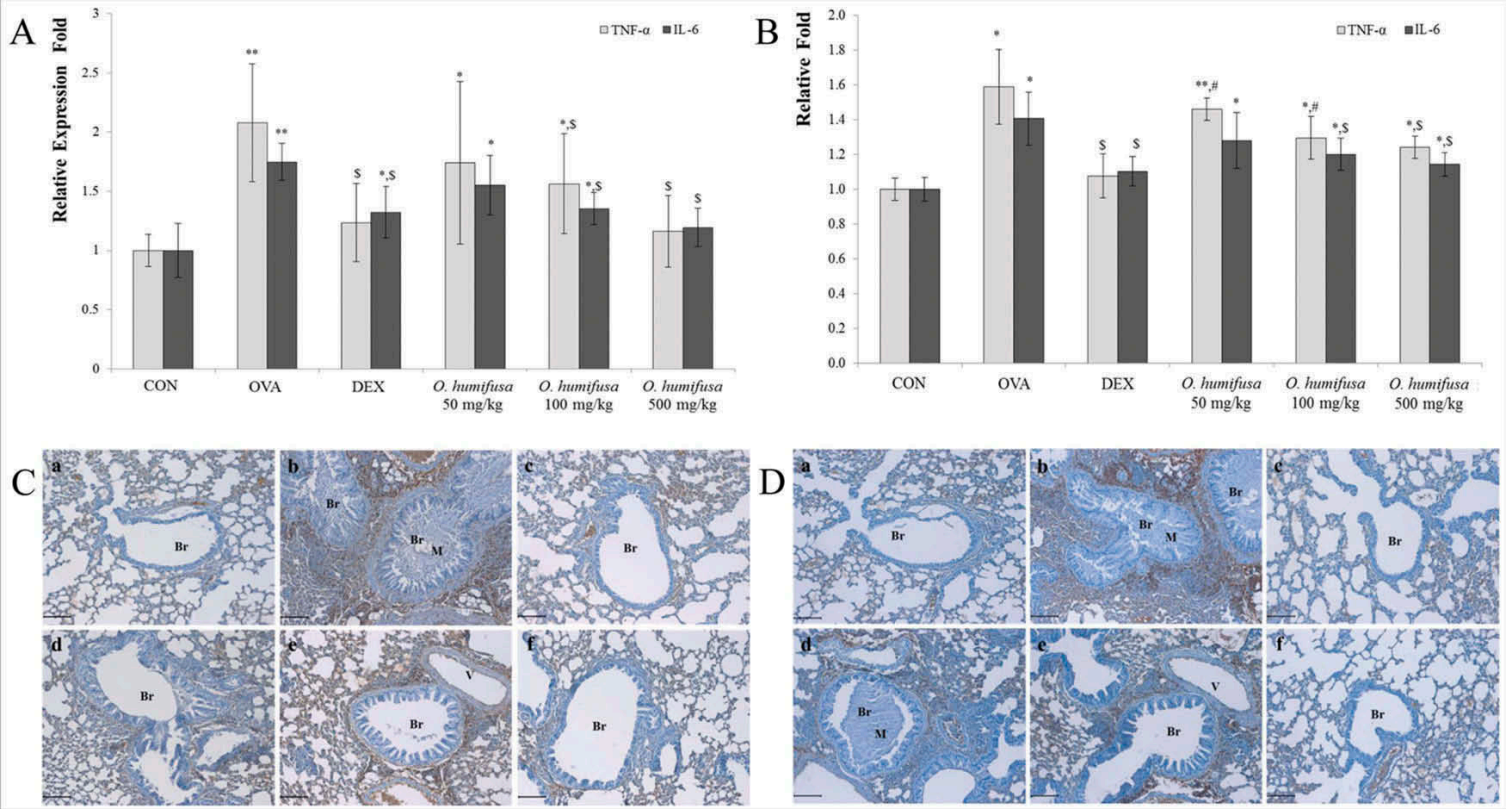
*O. humifusa* reduced gene expression and protein levels of Th17-related cytokines such as TNF-α and IL-6. *O. humifusa* down-regulated the expression of TNF-α and IL-6, as confirmed by RT-PCR (A), and their protein levels as assessed by ELISA (B) and IHC (C) in lung tissues; several were reduced in a dose-dependent manner. N = 8. Scale bar: 100 µm. Br, bronchiole; V, vessel; M, mucous; a, vehicle control; b, asthma induction; c, dexamethasone; d, 50 mg/kg/day *O. humifusa*; e, 100 mg/kg/day *O. humifusa*; f, 500 mg/kg/day *O. humifusa*. *p < 0.05 vs. control; **p < 0.001 vs. control; ^$^ *p* < 0.05 vs. asthma induction; ^#^ *p* < 0.05 vs. dexamethasone.

TNF-α gene expression was upregulated by OVA treatment, an effect that was reversed by dexamethasone treatment to levels observed in the control group. *O. humifusa* treatment downregulated TNF-α gene expression in a dose-dependent manner (Figure 5(a )); a similar pattern was observed for the IL-6 gene. Expression of TNF-α and IL-6 genes after treatment with 500 mg/kg *O. humifusa* was similar to that of the dexamethasone treatment group. Although *O. humifusa* suppressed both TNF-α and IL-6 gene expression, it was more effective at suppressing IL-6 expression (Figure 5(b,d)). TNF-α and IL-6 were dose-dependently downregulated by *O. humifusa* treatment; however, the expression of IL-6 was similar to that observed in the dexamethasone treatment group after treatment with 100 and 500 mg/kg *O. humifusa*.

## Discussion

Asthma is initially induced by antigen-presenting cells (APCs) that recognize a repeated allergen such as pollen, pet dander, dust mites, tobacco smoke, and environmental pollutants. For several decades, studies have attempted to elucidate the relationship between IgE and type 1 allergies, which are representative of asthma etiology [31,32]. The results of the present study suggest that reduced IgE expression regulates ovalbumin-induced asthma. Further, the balance between Th1 and Th2 activity is important for maintaining immunological homeostasis; an imbalance may result in the induction of various diseases, including asthma [33]. In asthmatic patients [1], Th2 cells recognize APCs and release cytokines such as IL-4 and IL-13, which stimulate class switching in B cells that produce IgE [31]. Changes in IFN-γ levels are related to IgE modulation [34]; under most known conditions of asthma induction, IgE levels increase [8].

In asthma, Th2-related cytokines such as IL-4 [35–37], IL-5 [38], and IL-13 [35,37] significantly increase; the development of antibodies against Th2-related cytokines is an active field of research [39]. Changes in Th17 cells correlate with asthma induction, making it important to analyze Th-17 proteins such as TNF-α, IL-6, and IL-1β [40]. We previously reported a correlation between TNF-α gene expression [41] and protein levels [42] and the induction of asthma. TNF-α has a chemoattractant effect on neutrophils and eosinophils [17]. Although an increase in eosinophil levels is observed in many asthma patients, other patients show a surge in neutrophils [30]. As shown in Figure 1(b), *O. humifusa* may regulate neutrophil levels, which are related to TNF-α functions (Figure 5(a,b,d)).

Both IL-6 and IL-13 levels are usually elevated in asthma patients and are presumed to be closely correlated [19]; we found that IL-6 levels were similar to IL-13 levels, supporting this idea. IL-13 and IL-6 levels after treatment with 500 mg/kg *O. humifusa* were notably lower than their levels after dexamethasone treatment.

**Figure 6.**
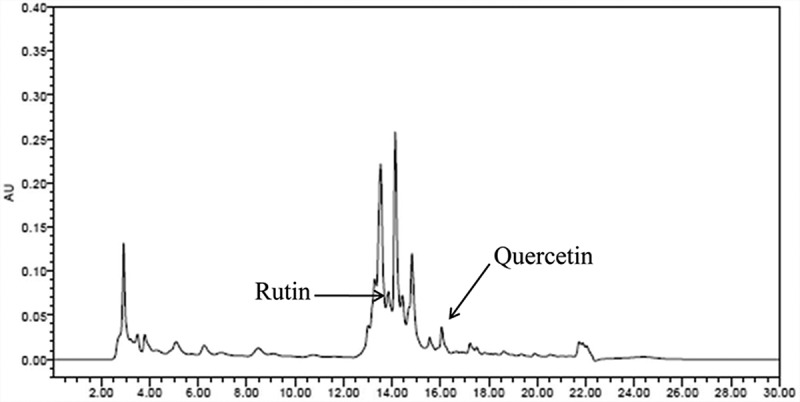
HPLC analysis of *O. humifusa*. We identified rutin (0.38 ± 0.005% at 13.85 min retention time) and quercetin (0.1 ± 0.001% at 16.05 min retention time) as components of *O. humifusa.*

To our knowledge, this is the first study to report the identification of active compounds from the *O. humifusa* leaf. We identified active compounds related to anti-asthma effects in the *O. humifusa* leaf using HPLC analysis, including bioactive markers related to anti-asthmatic activity such as rutin (0.38%, w/w) and quercetin (0.1%, w/w) (Figure 6).

We identified rutin and quercetin as two bioactive compounds found in the *O. humifusa* leaf. Cho et al. suggested the possibility of anti-inflammatory effect of ethylacetate fraction from *O. humifusa* using macrophage cells such as Raw 264.7 and identified the quercetin as one of the key components in *O. humifusa* [43]. Karym et al. reported that *Opuntia humifusa-indica* contains rutin in the range of 0.002–0.02% (w/w). Similary, we identified rutin in O. humifusa (0.38%), and rutin content in *O. humifusa* was 19 times higher than that of *O. ficus-indica* [44]. Cho et al. reported that *O. humifusa* leaf has an anti-inflammatory effect, and quercetin was reported as one of the key marker. In the present study, we have reported that *O. humifusa* leaf has anti-asthmatic activity and rutin and quercetin as biomarkers. Rutin and quercetin have been reported to contribute to anti-inflammatory efficacy as well as anti-asthmatic efficacy. Jung et al. [45] studied the effects of rutin and quercetin on asthmatic responses in OVA-sensitized, conscious guinea pigs. Rutin and quercetin (7.5 mg/kg, oral administration) notably and dose-dependently inhibited airway resistance in both immediate-phase and late-phase responses. At a dose of 15 mg/kg, rutin and quercetin also inhibited production of histamine, phospholipase 2, and erythropoietin. Thus, rutin and quercetin may be beneficial compounds for treating asthma. Therefore, if the effective dosage for rutin and quercetin in guinea pigs is 7.5 mg/kg/day, the human equivalent dosage is 97 mg/60 kg/day (*per os*). Extracts of *O. humifusa* showed anti-asthmatic effects at two evaluated doses and significantly suppressed the production of IL-4 and IL-13 at a dose of 500 mg/kg. We calculated the optimal oral intake of *O. humifusa* extract using the conversion factor between human and mouse (12.33). Therefore, if the effective dosage for mice is 500 mg/kg/day, the human equivalent dosage is 2425 mg/60 kg/day as extract or 10 and 2.5 mg/60 kg/day for rutin and quercetin, respectively. Other pharmaceutically active compounds other than rutin and quercetin may also be involved in the anti-asthmatic effects of *O. humifusa* and may have synergistic effects. Although *O. humifusa* has been studied for its biological effects in the treatment of diseases such as pancreatitis, cancer, and diabetes, nothing is known about the anti-asthmatic constituents of *O. humifusa* [24,46,47]. In the present study, we report anti-asthmatic effects and related constituents of *O. humifusa* for the first time. Further studies are needed to better understand the active constituents and pharmacological properties of *O. humifusa*.

*O. humifusa* effectively suppressed OVA-induced asthma by modulating Th1-/Th2-/Th17-related cytokines; in particular, Th2-related cytokines such as IL-4 and IL-13 were downregulated. *O. humifusa* prevented pulmonary morphological changes such as mucous hypersecretion, epithelial hyperplasia, and inflammatory cell infiltration near bronchioles and vessels. Although there are biochemical constituents in *O. humifusa*, the anti-asthmatic effects may be derived from rutin and quercetin. We conclude that *O. humifusa* is a potential functional food.

## Supplementary Material

Supplementary_Figure_1.tif
