# Supplementary figures and images for: Opuntia humifusa modulates morphological changes characteristic of asthma via IL-4 and IL-13 in an asthma murine model
Source: Food Nutr Res. 2017 Oct 24;61(1):1393307. doi: 10.1080/16546628.2017.1393307 (PMC5678225; doi:10.1080/16546628.2017.1393307)

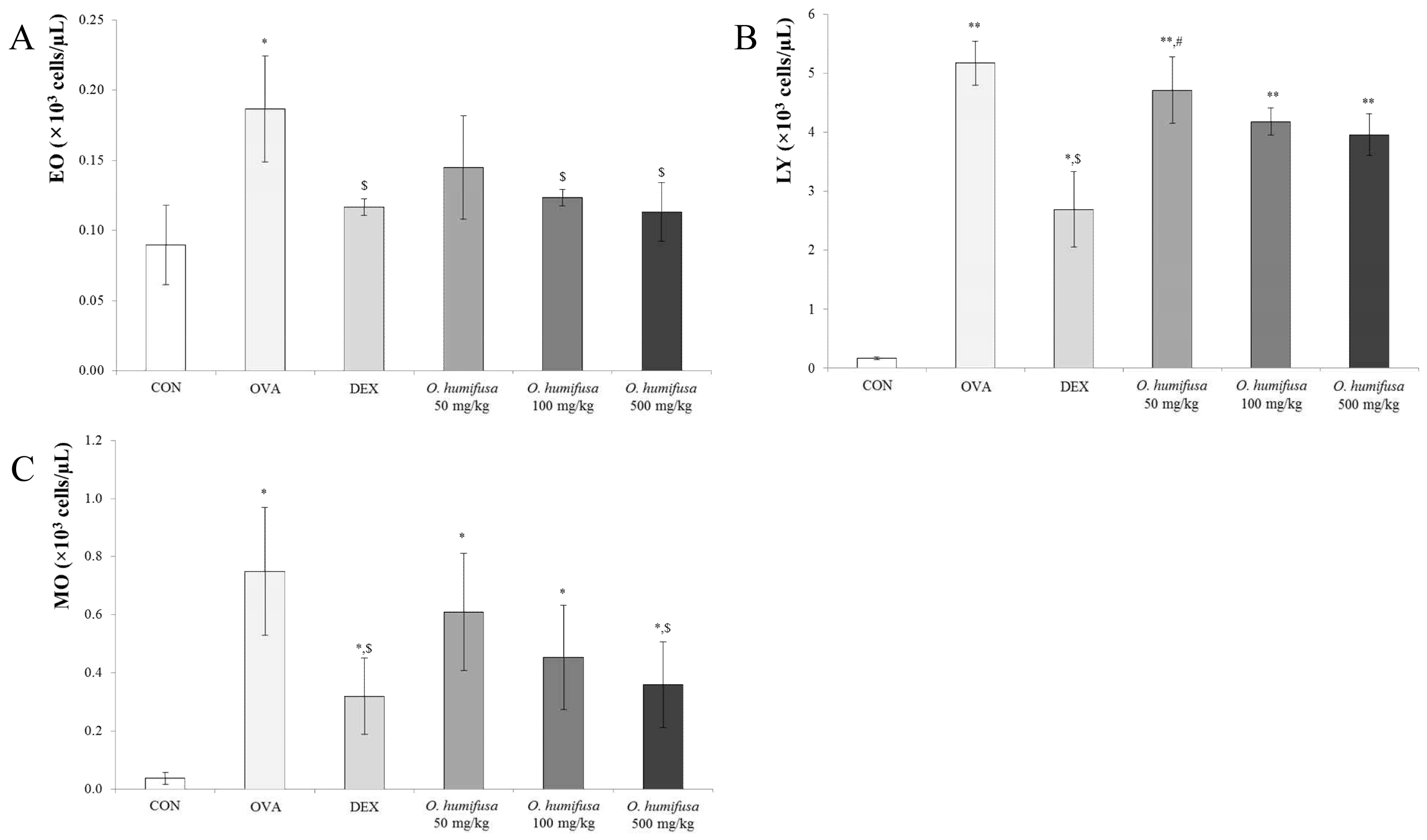

Supplement: Supplementary_Figure_1.tif [file ZFNR_A_1393307_SM2830.tif]
